# What is the relationship between contraceptive services and knowledge of abortion availability and legality? Evidence from a national sample of women and facilities in Ethiopia

**DOI:** 10.1093/heapol/czac103

**Published:** 2022-11-28

**Authors:** Linnea A Zimmerman, Celia Karp, Munir Kassa, Birikty Lulu, Mahari Yihdego, Selena Anjur-Dietrich, Assefa Seme, Solomon Shiferaw, Saifuddin Ahmed

**Affiliations:** Department of Population, Family, and Reproductive Health, Johns Hopkins Bloomberg School of Public Health, 615 N Wolfe Street, Baltimore, MD 21205, USA; Department of Population, Family, and Reproductive Health, Johns Hopkins Bloomberg School of Public Health, 615 N Wolfe Street, Baltimore, MD 21205, USA; Federal Ministry of Health, Ethiopia; Maternal, Child and Nutrition Directorate, Federal Ministry of Health, Ethiopia; Ethiopian Public Health Association, Ethiopia; Department of Population, Family, and Reproductive Health, Johns Hopkins Bloomberg School of Public Health, 615 N Wolfe Street, Baltimore, MD 21205, USA; School of Public Health, Addis Ababa University, Ethiopia; School of Public Health, Addis Ababa University, Ethiopia; Department of Population, Family, and Reproductive Health, Johns Hopkins Bloomberg School of Public Health, 615 N Wolfe Street, Baltimore, MD 21205, USA

**Keywords:** Abortion, family planning, contraception, legality

## Abstract

In Ethiopia, abortions are legal for minors and for rape, incest, foetal impairment or maternal disability. Knowledge of abortion legality and availability is low, and little effort has been made to disseminate this information for fear of invoking anti-abortion sentiment; instead, systems rely on health providers as information gatekeepers. This study explores how exposure to and interaction with family planning service delivery environment, specifically (1) availability of contraceptive and facility-based abortion services within 5 km of one’s residence and (2) contact with a health provider in the past 12 months, relate to women’s knowledge of the legality of accessing abortion services and of where to access facility-based abortion services. We used data from a nationally representative sample of 8719 women in Ethiopia and a linked health facility survey of 799 health facilities. Our outcome of interest was a categorical variable indicating if a woman had (1) knowledge of at least one legal ground for abortion, (2) knowledge of where to access abortion services, (3) knowledge of both or (4) knowledge of neither. We conducted multilevel, multinomial logistic regressions, stratified by residence. Approximately 60% of women had no knowledge of either a legal ground for abortion or a place to access services. Women who visited a health provider or who were visited by a health worker in the past 12 months were significantly more likely to know about abortion legality and availability. There were no differences based on whether women lived within 5 km of a facility that offered contraception and abortion services. We find that health workers are likely valuable sources of information; however, progress to disseminate information may be slowed if it relies on uptake of services and limited outreach. Efforts to train providers on legality and availability are critical, as is additional research on knowledge dissemination pathways.

**Key messages**

- The majority of women in Ethiopia are not aware of either legal grounds for abortion or where to access facility-based abortion services.
- Greater contact with health system, and specifically, use of contraceptive services, was associated with increased knowledge of both abortion legality and availability.
- Fewer than half of women visited a health facility in the past 12 months, and only about 10% were visited by a health worker who talked to them about family planning.
- As a result of the widespread reluctance to promote information on abortion legality widely, health workers have become gatekeepers to abortion services and information, particularly in private and non-governmental organizations (NGO) facilities; low level of contact with the health system may thus leave women without sufficient knowledge of legality or availability of safe abortion services.

## Introduction

Unintended births in sexually active women can be avoided through primary prevention of unintended pregnancies using contraception, backed by secondary prevention using induced abortion. Approximately 25% of unintended pregnancies result from contraceptive failure, however, necessitating the continued need for access to safe abortion services, even as contraceptive services expand (Sully *et al.*, 2020). While primary and secondary prevention are conceptualized as complementary, and both contraception and abortion have been identified as critical to achieving universal access to comprehensive sexual and reproductive health services in Africa (The African Union Commission, n.d.), the moral, political and legal challenges in providing safe abortion care often lead to separation of services.

Of the 121 million unintended pregnancies that occurred each year between 2015 and 2019, approximately 73 million ended in abortion (Bearak *et al.*, 2020). Despite the ubiquity of abortion across the globe, almost 700 million women live in countries where abortion is restricted or illegal (Center for Reproductive Rights, 2021). In these contexts, contraceptive services are generally the only legal means through which sexually active women can prevent an unintended birth. Global health funding restrictions imposed by high-income government donors, such as the Global Gag Rule (GGR) in the USA which prevents foreign organizations that receive US global health assistance from providing information, referrals or services for legal abortion even if they do not use US funds, further disrupt the provision of safe abortion care and public health systems (Ahmed, 2020). While legal constraints prevent women from accessing safe abortion services, they have little influence on women’s use of abortion as a secondary strategy to prevent an unintended birth, with comparable incidence rates in liberal and restrictive legal contexts (Sedgh *et al.*, 2016). These restrictions, however, significantly affect abortion-related morbidity and mortality, with higher morbidity and mortality rates associated with unsafe abortion (Ganatra *et al.*, 2017).

Beyond legal restrictions, access to safe abortion is conditioned on demand and supply factors. On the demand side, women’s knowledge of abortion legality and of abortion service availability informs their health-care-seeking decisions (Assifi *et al.*, 2016; Bell *et al.*, 2021; O’Connell *et al.*, 2022). Many women are unaware of their legal right to access abortion services (Assifi *et al.*, 2016; Sheehy *et al.*, 2021; O’Connell *et al.*, 2022), which may increase their reliance on unsafe measures (Banerjee *et al.*, 2012; Atakro *et al.*, 2019; Chemlal and Russo, 2019). On the other hand, the legality of abortion often fails to translate into accessible, high-quality and confidential services, especially at the primary care level (Banerjee *et al.*, 2012; Culwell and Hurwitz, 2013; Chemlal and Russo, 2019). The establishment of the GGR and expansions that followed, including restrictions on the provision of information about where to seek abortion services, has had far-reaching effects on the health system (Vernaelde, 2022). Even in settings where abortion is legal, clients have been unable to receive and providers have been unable to deliver comprehensive sexual and reproductive health services due to funding restrictions, while organizations have been reluctant to receive foreign assistance funds for fear of abrupt changes in policy (PAI, 2019).The GGR has also resulted in a ‘chilling’ effect, wherein providers fear running afoul of US guidelines and thus limit the provision of counselling on contraception care, even when these services are still allowable (Ahmed, 2020; Vernaelde, 2022).

Conversely, contraceptive services are more widely available to women, and though by no means universally accessible, significant global commitments have been made in recent years to improve access to and voluntary use of contraceptive methods (Brown *et al.*, 2014). Surveys across multiple settings have found generally high knowledge of both contraceptive methods and sources (Kennedy *et al.*, 2011; Blackstone *et al.*, 2017), and while lack of knowledge of or access to services is among reasons given for either contraceptive non-use or discontinuation, it is generally not among the most commonly reported (Sedgh and Hussain, 2014; Moreira *et al.*, 2019). Previous research has found mixed evidence that distance to health services affects contraceptive utilization, with some studies finding that increased distance and/or lower density of health services reduce the odds of contraceptive use (Ettarh and Kyobutungi, 2012; Skiles *et al.*, 2015; Shiferaw *et al.*, 2017; Wang and Mallick, 2019) while others finding a limited relationship between facility distance and health-care utilization (Heard *et al.*, 2004; Zimmerman *et al.*, 2019). If contraception and abortion are considered primary and secondary prevention within the same continuum of services, contraceptive services may play a critical role in increasing women’s knowledge of and access to safe abortion services. Despite this conceptual linkage, few studies have examined the relationship between access to and use of contraceptive services and women’s knowledge of abortion availability or legality, particularly in low-resource contexts where access to such care is limited.

### Family planning in Ethiopia

In Ethiopia, abortion is still considered illegal within the country’s Criminal Code; however, the legal status for abortions was broadened in 2005 to allow exemptions for minors and in cases of rape or incest, foetal impairment or maternal disability (FMOH, 2005). Although abortions that fall outside of these criteria are still considered illegal, a woman’s report of incest or rape, without police or other’s confirmation, is sufficient to secure an abortion, as is self-reported age or a broad range of medical indications, including self-reported threats to mental and physical well-being (Blystad *et al.*, 2019). Although the proportion of women accessing facility-based abortion services increased from 27% to 53% since the law was amended (Moore *et al.*, 2016), several studies indicate limited awareness of the legality and availability of safe abortion services in Ethiopia (Geleto and Markos, 2015; Bantie *et al.*, 2020; Sheehy *et al.*, 2021; O’Connell *et al.*, 2022). Despite low knowledge, there are relatively few efforts to widely disseminate language on the legal protections surrounding abortion to avoid provoking backlash against abortion protections (Blystad *et al.*, 2019; Tadele *et al.*, 2019). Efforts to promote access to safe abortion services have largely focussed on expanding facility readiness, strengthening the capacity of mid-level providers and relying on NGOs to offer and provide direct services (FMOH, 2006; Blystad *et al.*, 2019; Fekadu *et al.*, 2022). Due to the lack of transparency and confusion surrounding the legal code, safe abortion services are frequently left to the discretion of health providers (Blystad *et al.*, 2019; Fekadu *et al.*, 2022), and evidence suggests that there is widespread belief in the right of refusal, despite no such exception existing (Fekadu *et al.*, 2022). Qualitative research indicates that health providers play a critical role in not only offering abortion services but also disseminating information about both legality and availability, resulting in patchwork implementation that differs significantly by region and residence (Gebrehiwot *et al.*, 2016; Tadele *et al.*, 2019). To date, evidence on these associations has been largely generated from qualitative research.

In addition to expanding legal protections for abortion services, the Federal Ministry of Health (FMoH) has invested considerably in the promotion and provision of contraceptive services in recent years (Olson and Piller, 2013), including being a signatory to both FP2020 and FP2030 initiatives that aimed to increase access to and use of family planning (FP) services (FP2030, 2018). Modern contraceptive use among married women of reproductive age has increased substantially in Ethiopia over the last two decades, rising from 8% in 2000 to 36% in 2020 (PMA Ethiopia, 2021). The majority of modern contraceptive users (77.4%) report accessing their services directly from public health providers (PMA2020, 2018). In addition to health facilities, health extension workers (HEWs) play an important role in expanding the reach of and access to health services among people living in Ethiopia’s rural communities (Wang *et al.*, 2016). To address a shortage of health providers (one physician for every 9979 people, one nurse for every 1705 and one midwife for every 5491 people) (Fact Sheets| MINISTRY OF HEALTH - Ethiopia, n.d.), Ethiopia launched the Health Extension Program in 2004 that included the creation of the HEW position. HEWs are community-based health providers who provide health education and basic curative services, including contraception. Two HEWs are generally assigned to each kebele, which is the lowest administrative unit, with an average of 1000 households or 5000 people (Wang *et al.*, 2016). Although HEWs do not provide abortion services directly, dissemination of information on abortion safety and legality is part of their mandate (Federal Democratic Republic of Ethiopia Ministry of Health, 2011). HEWs have additionally been central to enhancing coverage of Ethiopia’s FP program through the provision of a range of contraceptive methods and information about these methods, even among women who choose not to use contraception (Sedlander *et al.*, 2018). While increased use of contraception among women in rural Ethiopia has been attributed, in part, to HEWs, no studies have explored whether engagement with an HEW encompasses broader transmission of sexual and reproductive health information, including legality and accessibility of abortion services.

### Objective

This study explores how exposure to and interaction with FP service delivery environment, specifically (1) availability of contraceptive and facility-based abortion services within 5 km of one’s residence and (2) contact with a health provider, inclusive of facility-based providers at either public or private clinics and HEWs, in the past 12 months, relate to women’s knowledge of legality of accessing abortion services and knowledge of where to access facility-based abortion services in Ethiopia, accounting for women’s socio-demographic characteristics. We hypothesize that women who live closer to facilities that offer comprehensive FP services, including both contraceptive and safe abortion care, and those who have discussed FP with a health provider who visited the home or a facility-based provider in the past 12 months will be more likely to know about both the legality of abortion services and where a woman can access abortion care in their communities.

## Materials and methods

### Data sources

This cross-sectional study uses data from Performance Monitoring for Action (PMA)-Ethiopia, a 5-year (2019–2023) research partnership between Addis Ababa University (AAU), the Ethiopian FMoH and the Johns Hopkins Bloomberg School of Public Health (JHSPH). PMA-Ethiopia generates cross-sectional and longitudinal data on a range of reproductive, maternal and newborn health indicators (Zimmerman *et al.*, 2020). Data are collected from women, households and service delivery points (SDPs) that offer maternal and reproductive health services to inform policies and priorities at national and regional levels.

This analysis uses two data sources from PMA-Ethiopia, the cross-sectional, nationally representative sample of women aged 15–49 years (Addis Ababa University School of Public Health and The Bill & Melinda Gates Institute for Population and Reproductive Health at The Johns Hopkins Bloomberg School of Public Health, 2020) and data from the cross-sectional SDP survey (Addis Ababa University School of Public Health; and the Bill & Melinda Gates Institute for Population and Reproductive Health at the Johns Hopkins Bloomberg School of Public Health, 2019) collected between October and November 2019. National cross-sectional data included both household and female surveys. Enumeration areas (EAs), groupings of approximately 200 households, were selected with probability proportional to size within regional and residential strata, using the national census as the sampling frame. Following a census and listing, 35 households were randomly selected within each EA; all households within a selected EA were eligible for the household survey. Women were eligible to participate if they were aged between 15 and 49 years, slept in the selected household the night prior or who were usual members of the household and were willing and able to provide informed consent.

The SDP survey (herein referred to as the ‘facility survey’) was conducted among public and private facilities that served the selected EAs. Public facilities were included if they were a health post, health centre or hospital. A maximum of three private SDPs, including health clinics and health centres that offered maternal health services and pharmacies or drug vendors that provided reproductive health commodities, within the kebele (the smallest administrative unit in Ethiopia) in which the EA was located were randomly selected for interview. Data about facility readiness to offer essential health services, including provision of abortion services, were collected by trained resident interviewers using mobile phones equipped with Open Data Kit Software (Open Data Kit, Seattle, WA, USA). Study procedures, detailed elsewhere including additional information on consent procedures and ethical concerns, were approved by the Institutional Review Boards at Johns Hopkins School of Public Health and AAU (Zimmerman *et al.*, 2020).

### Measures

Our primary dependent variable was a categorical measure assessing women’s knowledge of legal grounds for abortion and knowledge of service availability. We defined respondents as having knowledge of legal grounds for abortion using two conditions: if (1) a woman said yes to the question ‘Do you know if there is a law on abortion in Ethiopia?’ and (2) if she spontaneously reported one or more of the conditions under which abortion is legal in Ethiopia to the question ‘Under which circumstances, it is legal to have an abortion in Ethiopia?’. Interviewers were trained to select one or more of the options that included rape, foetal impairment, risk to the life of the mother or foetus or if the mother is unable to raise the child due to physical or mental infirmity. We chose to define ‘knowledge’ based on identifying a single exemption, rather than all exemptions, as the percentage of women with knowledge of all legal exemptions based on previous research were less than 5% of women (Sheehy *et al.*, 2021). Abortion is also legal in Ethiopia for women under age of 18 years; however, due to a survey programming error, this legal ground was not assessed in the questionnaire. Women could also respond that they knew that there was a law about abortion and that abortion was not legal under any circumstances; these women were not categorized as having knowledge of the legal grounds for abortion. We measured women’s knowledge of where to access services through a single item, ‘Do you know where a woman can access facility-based abortion services in the community where you live?’ (Yes/no). Based on responses to these three questions, women were classified into one of the four categories: (1) no knowledge of either legality or service availability, (2) knowledge of legal grounds only, (3) knowledge of service availability only or (4) knowledge of legality and service availability.

Our key independent variables measured a woman’s exposure to and use of the contraceptive service environment, which we examined via three measures captured across the female and facility surveys. First, using the female survey, we assessed two measures: (1) whether the respondent had been visited by a health provider who discussed FP^1^ in the last 12 months (yes/no) and (2) whether the respondent had visited a health facility in the past 12 months and spoken to someone about FP (categorical: no visit, visit but no FP discussion and visit and FP discussion). Second, using data from the facility survey, we assessed availability of facility-based contraceptive and abortion services for each woman in our sample. Specifically, we identified all facilities within a 5 km radius (geodetic distance) of the woman’s residence and defined a three-level categorical variable indicating availability of services: (1) no contraceptive or abortion services within 5 km, (2) contraceptive services only within 5 km and (3) contraceptive and abortion services available within 5 km. No facilities provided abortion without contraceptive services. Finally, we explored the role of women’s contraceptive use status, which we defined as either using or not using a modern contraceptive method at the time of the survey.

Due to significant differences in availability of services and wealth distributions between urban and rural areas (Table 1), all analyses were stratified by residence. Analyses adjusted for a number of additional socio-demographic characteristics and potential confounders that we hypothesized to be associated with exposure to the FP service environment and knowledge of abortion legality and services, including women’s age (5-year age groups), marital status (married/in-union, not in union), wealth quintiles, education (none, primary, secondary or above), parity (0, 1–2, 3–4 and 5+ children) and region. Given high correlation between age and parity (ρ > 0.65 across residence), age was excluded in final models. Wealth quintiles were heavily skewed in urban and rural areas when stratified (Table 1). For subsequent analyses, we thus created wealth quintiles specific to urban/rural women to assess the effect of wealth separately within each residence.

**Table 1.** Sample characteristics of women participating in the PMA-Ethiopia 2019 cross-sectional survey

	Total	Rural	Urban	*P*-value
		***N***		
	**8724**	**5870**	**2854**	
	***n* (%)**	***n* (%)**	***n* (%)**	
Knowledge				
Neither	5410 (62.0)	3940 (67.1)	1470 (51.5)	<0.001
Legal only	702 (8.1)	386 (6.6)	317 (11.1)	
Source only	1398 (16.0)	926 (15.8)	472 (16.5)	
Legal and source	1214 (13.9)	619 (10.5)	595 (20.9)	
Facilities offering FP in 5 km				
None	626 (7.2)	626 (10.7)	0 (0.0)	<0.001
Offer contraception only	4159 (47.6)	3716 (63.3)	434 (15.2)	
Offer contraception and abortion	3947 (45.2)	1528 (26.0)	2420 (84.8)	
Facility visit				
None	4160 (47.7)	3010 (51.3)	1150 (40.3)	<0.001
Visit, no contraception	3565 (40.9)	2167 (36.9)	1398 (49.0)	
Visit, with contraception	999 (11.5)	694 (11.8)	305 (10.7)	
Visited by a health worker	813 (9.3)	580 (9.9)	234 (8.2)	0.21
Contraceptive use	2253 (25.8)	1434 (24.4)	819 (28.7)	0.03
Age				
15–19	1936 (22.2)	1314 (22.4)	622 (21.8)	<0.001
20–24	1522 (17.5)	890 (15.2)	633 (22.2)	
25–29	1643 (18.8)	988 (16.8)	655 (23.0)	
30–34	1142 (13.1)	785 (13.4)	357 (12.5)	
35–39	1133 (13.0)	865 (14.7)	268 (9.4)	
40–44	778 (8.9)	592 (10.1)	186 (6.5)	
45–49	570 (6.5)	436 (7.4)	134 (4.7)	
Married	5756 (66)	4202 (71.6)	1554 (54.5)	<0.001
Wealth				
Lowest	1678 (19.2)	1655 (28.2)	24 (0.8)	<0.001
Lower	1678 (19.2)	1607 (27.4)	71 (2.5)	
Middle	1693 (19.4)	1561 (26.6)	132 (4.6)	
Higher	1688 (19.4)	940 (16.0)	748 (26.2)	
Highest	1986 (22.8)	106 (1.8)	1879 (65.9)	
Education				
None	3303 (37.9)	2867 (48.9)	436 (15.3)	<0.001
Primary	3197 (36.7)	2249 (38.3)	947 (33.2)	
Secondary +	2220 (25.5)	750 (12.8)	1470 (51.5)	
Parity				
0	2823 (32.4)	1578 (26.9)	1245 (43.6)	<0.001
1–2	2278 (26.1)	1319 (22.5)	960 (33.6)	
3+	3621 (41.5)	2972 (50.6)	649 (22.7)	
Region				
Tigray	556 (6.4)	369 (6.3)	186 (6.5)	<0.001
Amhara	2043 (23.4)	1501 (25.6)	542 (19.0)	
Oromiya	3297 (37.8)	2404 (40.9)	894 (31.3)	
SNNP	1680 (19.3)	1226 (20.9)	454 (15.9)	
Addis	538 (6.2)	0 (0.0)	538 (18.9)	
Others	698 (7.0)	370 (6.3)	239 (8.4)	

Of 8976 eligible women who slept in the house the night before (*de facto* residents), 8839 completed the interview, with a response rate of 98.4%. For this analysis, we dropped 120 women who were missing outcome data (described further later) for a total population of 8719 women (unweighted: *n* = 3738 urban and *n* = 4981 rural; weighted: *n* = 2854 urban and *n* = 5870 rural). Of 815 SDPs identified for the survey, 799 completed the interview, with a response rate of 98.0%. All observations were included.

### Analysis

We used design-based analysis to account for survey weighting due to differential probability of selection and clustering of responses within EAs (Heeringa *et al.*, 2017). Exploratory analyses assessed the distributions of the key outcome and predictor variables and women’s socio-demographic characteristics. We then assessed outcomes by urban and rural strata separately and tested for differences using Pearson chi-square statistics with the Rao and Scott second-order correction. Finally, we used stratified multinomial multilevel regression models, with EA as the second level, to estimate the relative risk ratios (RRRs) of having knowledge of legal grounds only, service availability only or knowledge of both, relative to having no knowledge of either. Models 1 and 3 (rural and urban, respectively) included only covariates that related to exposure to the FP environment, specifically distance to services, visit to a health facility, visits from a health worker, modern contraceptive method use and region. Models 2 and 4 (rural and urban, respectively) additionally adjusted for relevant individual-level socio-demographic characteristics. We tested for shared versus separate random effects using Akaike Information Criterion and Bayesian Information Criterion and treated random effects as separate but correlated. All analyses were conducted using Stata 16.1 (College Station, TX, USA).

## Results

Sample characteristics are presented in Table 1. Weighted counts and percentages are presented. The *P*-value from the adjusted Pearson chi-square testing for differences in distributions between urban and rural samples is shown. There were significant differences between urban and rural women across almost every outcome and socio-demographic characteristic. Urban women were younger, less likely to be married, more educated and of lower parity. Rural women were significantly poorer, with 1.8% of rural women living in the wealthiest quintile relative to 65.9% of urban women. Urban women had greater knowledge of abortion legality and availability, although more than half of women in both urban and rural areas did not have knowledge of either. The majority of urban women (84.9%) lived within 5 km of a health provider who provided both abortion and contraceptive services, while only 26.0% of rural women had similar access.

Table 2 shows the percentages of women in each abortion knowledge category by background characteristic, stratified by urban and rural residence. Among rural women, there were significant differences by all characteristics, except marital status. Among urban women, all differences were significant other than by distance of a health facility that offered contraceptive and/or abortion services, by whether the woman was visited by a health worker or by marital status.

**Table 2.** Bivariate distributions of knowledge of abortion legality and availability by background characteristic, stratified by rural and urban residence. PMA-Ethiopia 2019 cross-sectional survey

	Rural					Urban				
	None	Legal only	Source only	Both		None	Legal only	Source only	Both	
			**Row %**		***P*-value**			**Row %**		***P*-value**
Facilities offering FP in 5 km										
None	80.5	3.3	11.2	4.9	0.01					
Offer contraception only	69.6	7.6	13.5	9.4		61.7	6.7	16.5	15.1	0.34
Offer contraception and abortion	55.7	5.4	23.2	15.7		49.7	11.9	16.6	21.9	
Facility visit										
None	72.1	6.8	13.3	7.9	<0.001	58.5	9.8	14.7	17.1	0.008
Visit, no contraception	62.5	6.1	18.6	12.9		47.3	11.2	18.7	22.9	
Visit, with contraception	60.1	7.3	17.7	15.0		44.7	15.4	13.9	26.0	
Visited by a health worker										
No	67.6	6.1	16.1	10.2	0.005	52.2	11.0	16.4	20.4	0.21
Yes	62.9	10.8	12.7	13.6		43.8	12.5	18.2	25.5	
MCP										
Non-user	69.0	6.0	14.9	10.1	0.001	54.0	10.6	16.1	19.2	0.002
User	61.2	8.3	18.6	11.9		45.2	12.2	17.6	24.9	
Age										
15–19	66.0	7.2	16.2	10.5	0.01	63.1	9.6	15.0	12.3	<0.001
20–24	61.3	8.2	17.4	13.1		47.2	14.3	18.7	19.8	
25–29	63.0	9.0	15.8	12.3		47.1	11.7	16.9	24.3	
30–34	70.1	4.7	14.8	10.3		48.2	9.9	15.8	26.2	
35–39	70.5	5.6	15.3	8.6		46.6	10.6	17.5	25.3	
40–44	70.8	3.8	15.3	10.2		54.0	10.2	13.6	22.1	
45–49	74.4	4.8	14.4	6.3		54.5	5.8	15.9	23.7	
Married										
No	65.3	6.7	15.9	12.1	0.30	52.5	11.6	16.4	19.5	0.46
Yes	67.9	6.5	15.7	9.9		50.7	10.6	16.7	22.0	
Wealth										
Lowest	76.0	4.2	11.3	8.5	0.003	67.5	7.8	15.7	9.0	<0.001
Lower	71.7	5.5	14.5	8.4		57.0	9.5	14.7	18.9	
Middle	67.9	6.2	18.0	8.0		46.7	12.5	16.3	24.4	
Higher	62.8	7.3	18.1	11.8		39.3	13.7	19.5	27.4	
Highest	56.3	10.0	17.0	16.7		38.3	13.7	17.8	30.2	
Education										
None	75.0	3.9	14.0	7.0	<0.001	73.2	7.3	11.0	8.5	<0.001
Primary	65.0	8.3	15.6	11.1		60.3	9.1	16.5	14.1	
Secondary +	43.2	11.4	23.2	22.2		39.4	13.5	18.2	28.9	
Parity										
0	63.4	7.4	17.7	11.5	<0.001	53.0	12.7	16.6	17.7	0.004
1–2	61.7	9.1	15.7	13.5		46.3	10.8	16.4	26.4	
3+	71.5	5.0	14.8	8.7		56.3	8.4	16.6	18.7	
Region										
Tigray	44.7	3.8	24.2	27.4	0.003	27.6	4.1	33.5	34.7	0.02
Amhara	65.9	6.5	16.9	10.7		50.9	10.0	15.2	23.9	
Oromiya	66.6	9.0	15.8	8.5		55.7	13.7	14.2	16.3	
SNNP	69.4	3.7	15.7	11.3		57.8	7.3	15.8	19.1	
Addis						39.4	15.8	18.2	26.6	
Others	90.2	3.3	2.9	3.6		70.7	6.1	12.8	10.4	

Table 3 shows the results of Model 1, which includes only service delivery environmental factors in rural areas. Among rural women, relative to women who did not visit a health facility in the past 12 months, those who visited a health facility in the past 12 months and discussed FP during the visit were more likely to know at least one legal ground for abortion [RRR: 1.66, 95% confidence interval (CI): 1.10–2.51], more likely to know a place to access facility-based abortion services (RRR: 1.67, 95% CI: 1.20–2.33) and more likely to know both a legal ground and a place to access services (RRR: 2.06, 95% CI: 1.44–2.96). Women who visited a health facility but did not speak with a provider about FP were no more likely to know a legal ground for services but were more likely to know a place to access facility-based abortion services (RRR: 1.31, 95% CI: 1.04–1.64) and have knowledge of both legal grounds and facility services (RRR: 1.56, 95% CI: 1.20–2.02). Relative to women in Oromiya, women living in smaller regions of Ethiopia (‘Other’ regions) were significantly less likely to know only a source of facility-based abortion services (RRR: 0.15, 95% CI: 0.06–0.40), and women in Tigray were significantly more likely to know both a legal ground and a source of services (RRR: 7.16, 95% CI: 1.80–28.55).

**Table 3.** Multilevel multinomial logistic regression results among rural women. PMA-Ethiopia 2019 cross-sectional survey

	Legal only			Source only			Both		
	RRR	95% CI		RRR	95% CI		RRR	95% CI	
Facilities offering FP in 5 km (ref: none)									
Offer contraception only	1.14	0.49	2.70	0.96	0.47	1.97	1.00	0.45	2.26
Offer contraception and abortion	1.50	0.61	3.71	1.72	0.80	3.71	2.07	0.86	5.00
Facility visit (ref: none)	0.00			0.00			0.00		
Visit, no contraception	1.13	0.83	1.53	1.31*	1.04	1.64	1.56^***^	1.20	2.02
Visit, with contraception	1.66*	1.10	2.51	1.67^**^	1.20	2.33	2.06^***^	1.44	2.96
Visited by a health worker (ref: no)	0.00			0.00			0.00		
Yes	1.41	0.95	2.10	0.84	0.59	1.21	1.37	0.97	1.94
MCP (ref: non-user)	0.00			0.00			0.00		
User	1.11	0.82	1.51	1.23	0.96	1.57	1.04	0.79	1.36
Region (ref: Oromiya)	0.00			0.00			0.00		
Tigray	0.61	0.19	1.98	2.94	0.96	8.95	7.16^***^	1.80	28.55
Amhara	0.67	0.28	1.61	1.14	0.47	2.78	1.71	0.56	5.21
SNNP	0.44	0.18	1.09	1.18	0.48	2.89	1.25	0.40	3.91
Others	0.45	0.18	1.10	0.15^***^	0.06	0.40	0.38	0.12	1.25
M1 EA ID	2.72			2.72			2.72		

* *P* < 0.05

** *P* < 0.01

*** *P* < 0.001.

The results for Model 2, after adjusting for relevant socio-demographic characteristics, are presented in Table 4. In general, relationships remain unchanged; however, women who were visited by a health worker who spoke with them about FP were significantly more likely to know a legal ground for abortion and know both a legal ground and a source of abortion services (adjusted Relative Risk Ratio (aRRR): 1.64, 95% CI: 1.09–2.47 and aRRR: 1.51. 95% CI: 1.05–2.16, respectively). Among socio-demographic characteristics, only education was consistently related to knowledge, with increasing education significantly increasing the odds of knowing a legal ground, a source of abortion services or both.

**Table 4.** Adjusted multilevel multinomial logistic regression results among rural women. PMA-Ethiopia 2019 cross-sectional survey

	Legal Only			Source Only			Both		
	RRR	95% CI		RRR	95% CI		RRR	95% CI	
Facilities offering FP in 5 km (ref: no FP services)									
Offer contraception only	0.96	0.40	2.30	0.90	0.43	1.85	0.83	0.36	1.91
Offer contraception and abortion	1.30	0.52	3.25	1.60	0.73	3.49	1.78	0.72	4.38
Facility visit (ref: none)									
Visit, no contraception	1.08	0.78	1.50	1.36*	1.07	1.73	1.57^**^	1.19	2.07
Visit, with contraception	1.75*	1.13	2.71	1.78^**^	1.26	2.52	2.23^***^	1.51	3.27
Visited by a health worker (ref: no)									
Yes	1.64*	1.09	2.47	0.92	0.64	1.33	1.51*	1.05	2.16
MCP (ref: non-user)									
User	1.08	0.77	1.51	1.26	0.97	1.63	1.11	0.82	1.49
Region (ref: Oromiya)									
Tigray	0.62	0.19	1.99	2.91	0.96	8.78	6.48^**^	1.58	26.48
Amhara	0.72	0.31	1.71	1.15	0.48	2.78	1.89	0.61	5.90
SNNP	0.44	0.18	1.08	1.14	0.47	2.78	1.28	0.40	4.11
Other	0.45	0.18	1.11	0.15^***^	0.06	0.39	0.38	0.11	1.27
Marital status (ref: not married)									
Married	1.45	0.96	2.18	1.43*	1.05	1.96	0.94	0.67	1.32
Wealth (ref: lowest)									
Lower	1.02	0.62	1.70	1.31	0.92	1.89	0.62*	0.40	0.95
Middle	1.03	0.61	1.72	1.49*	1.02	2.17	0.67	0.43	1.05
Higher	1.03	0.61	1.73	1.41	0.96	2.08	0.99	0.64	1.54
Highest	1.26	0.72	2.20	1.25	0.80	1.94	1.20	0.73	1.97
Education (ref: none)									
Primary	2.48^**^	1.77	3.47	1.44^**^	1.11	1.86	2.19^***^	1.62	2.95
Secondary	4.41^**^	2.76	7.06	2.58^***^	1.78	3.72	6.90^***^	4.58	10.40
Parity (ref: 0)									
1–2	1.09	0.71	1.68	0.74	0.52	1.05	1.43	0.97	2.10
3+	0.67	0.43	1.05	0.68*	0.47	0.97	1.22	0.81	1.83

* *P* < 0.05

** *P *< 0.01

*** *P* < 0.001.

Table 5 shows the results of Model 3, which includes only service delivery environmental factors in urban areas. Among urban women, women who lived within 5 km of a facility that offered both contraception and abortion services were more likely to know a legal ground for abortion than women who lived within 5 km of facility that offered only contraceptive services (RRR: 2.34, 95% CI: 1.06–5.17). Patterns are similar to rural women, with visits to a health facility, both with and without speaking to a provider, being associated with significantly higher likelihood of knowing a place to access facility-based abortion services and knowing both a legal ground and a place to access services. Women who were visited by a health worker in the past 12 months who spoke to them about FP were more likely to know a legal ground (RRR: 1.77, 95% CI: 1.17–2.68), a source of facility-based abortions (RRR: 2.08, 95% CI: 1.48–3.02) or both (RRR: 1.74, 95% CI: 1.24–2.44). Women who were using a modern method of contraception were more likely to know both a legal ground and a source of facility-based abortions (RRR: 1.47, 95% CI: 1.18–1.83). Relative to women in Oromiya, women in Tigray had significantly higher relative risk of knowing only a source of abortion availability (RRR: 8.81, 95% CI: 3.34–23.23) or of knowing both a source and a legal ground (RRR: 6.94, 95% CI: 2.75–17.55).

**Table 5.** Multilevel multinomial logistic regression results among urban women, service delivery factors only. PMA-Ethiopia 2019 cross-sectional survey

	Legal only			Source only			Both		
	RRR	95% CI		RRR	95% CI		RRR	95% CI	
Facilities offering FP in 5 km (ref: contraception only)									
Offer contraception and abortion	2.34*	1.06	5.17	1.07	0.46	2.52	2.19	0.96	5.03
Facility visit (ref: none)									
Visit, no contraception	1.34*	1.03	1.76	1.63^***^	1.29	2.05	1.86^***^	1.50	2.31
Visit, with contraception	1.26	0.82	1.93	1.55*	1.06	2.27	2.42^***^	1.73	3.37
Visited by a health worker (ref: no)									
Yes	1.77^**^	1.17	2.68	2.08^**^	1.43	3.02	1.74^**^	1.24	2.44
MCP (ref: non-user)									
User	1.21	0.92	1.60	1.39^**^	1.09	1.76	1.47^***^	1.18	1.83
Region (ref: Oromiya)									
Tigray	0.52	0.20	1.36	8.81^***^	3.34	23.23	6.94^***^	2.75	17.55
Amhara	0.98	0.39	2.47	1.49	0.50	4.39	2.10	0.76	5.85
SNNP	0.62	0.25	1.53	1.05	0.37	3.02	1.02	0.37	2.81
Addis	1.64	0.77	3.51	1.95	0.78	4.89	2.35	0.99	5.61
Other	0.86	0.39	1.87	0.71	0.28	1.83	0.96	0.40	2.34

* *P* < 0.05

** *P* < 0.01

*** *P* < 0.001.

After adjustment for socio-demographic characteristics, these relationships were somewhat modified in Model 4 (Table 6). Distance to services was no longer significant with any outcome, while visiting a health provider who did not talk about FP was no longer related to knowing only a legal ground for abortion. Being visited by a health provider who discussed FP remained strongly associated with all outcomes, while being a contraceptive user increased the odds of knowing a source for facility-based services and knowing both a legal ground and a source. Increasing wealth significantly increases the odds of knowing only a legal ground for abortion or both a legal ground and a source of abortion services. Increasing education is positively associated with all outcomes, while increasing parity is related to knowing only a source of services and both a source and a legal ground.

**Table 6.** Adjusted multilevel multinomial logistic regression results among rural women. PMA-Ethiopia 2019 cross-sectional survey

	Legal only			Source only			Both		
	RRR	95% CI		RRR	95% CI		RRR	95% CI	
Facilities offering FP in 5 km (ref: contraception only)									
Offer contraception and abortion	1.84	0.84	4.03	1.01	0.44	2.32	1.75	0.79	3.88
Facility visit (ref: none)									
Visit, no contraception	1.29	0.97	1.72	1.52^***^	1.19	1.94	1.54^***^	1.22	1.95
Visit, with contraception	1.30	0.83	2.04	1.44	0.97	2.15	2.06^***^	1.44	2.95
Visited by a health worker (ref: no)									
Yes	1.99^**^	1.30	3.07	2.08^***^	1.42	3.05	1.76^**^	1.23	2.51
MCP (ref: non-user)									
User	1.24	0.90	1.70	1.32*	1.02	1.72	1.29*	1.01	1.65
Region (ref: Oromiya)									
Tigray	0.43	0.17	1.10	7.96^***^	3.11	20.39	5.89^***^	2.47	14.06
Amhara	1.18	0.48	2.92	1.59	0.56	4.55	2.53	0.97	6.62
SNNP	0.60	0.25	1.45	1.02	0.37	2.82	0.97	0.38	2.50
Addis	1.30	0.62	2.75	1.77	0.72	4.33	1.92	0.85	4.34
Other	0.89	0.42	1.90	0.72	0.29	1.79	1.01	0.44	2.32
Marital status (ref: not married)									
Married	1.02	0.73	1.42	0.96	0.73	1.27	0.90	0.69	1.17
Wealth (ref: lowest)									
Lower	1.57	0.97	2.56	1.04	0.71	1.51	1.71^**^	1.15	2.53
Middle	2.03^**^	1.22	3.39	1.17	0.78	1.75	2.24^***^	1.48	3.38
Higher	2.29^**^	1.34	3.91	1.42	0.93	2.15	2.31^***^	1.50	3.56
Highest	2.31^**^	1.33	4.00	1.21	0.77	1.90	2.71^***^	1.73	4.24
Education (ref: none)									
Primary	1.59	0.96	2.64	1.61*	1.12	2.33	2.08^***^	1.41	3.06
Secondary	4.57^***^	2.76	7.56	2.99^***^	2.03	4.35	7.33^***^	4.96	10.84
Parity (ref: 0)									
1–2	1.05	0.74	1.48	1.35	1.00	1.82	2.02^***^	1.53	2.67
3+	1.06	0.68	1.63	1.52*	1.06	2.17	2.48^***^	1.77	3.48

* *P* < 0.05

** *P* < 0.01

*** *P* < 0.001

## Discussion

We find that the majority of women in Ethiopia are not aware of either legal grounds for abortion or where to access facility-based abortion services. While greater contact with the health system, and specifically, use of contraceptive services, was associated with increased knowledge of both abortion legality and availability, fewer than half of women visited a health facility in the past 12 months and only about 10% were visited by a health worker who talked to them about FP. As a result of the widespread reluctance to promote information on abortion legality widely, health workers frequently serve as gatekeepers to abortion services and information, particularly in private and NGO facilities; low level of contact with the health system may thus leave women without sufficient knowledge of legality or availability of safe abortion services.

Overall, women demonstrated low levels of knowledge of abortion legality or availability. Approximately 30% of women did know where to access facility-based services, but of these, more than half did not know any legal ground for abortion services. These levels of knowledge align with previous studies that confirm low levels of abortion knowledge among women in Ethiopia (Geleto and Markos, 2015; Bantie *et al.*, 2020; Sheehy *et al.*, 2021; O’Connell *et al.*, 2022). Not knowing when or if abortion services can be offered legally and safely may significantly increase the risk of relying on unsafe methods for abortion (Banerjee *et al.*, 2012; Atakro *et al.*, 2019; Chemlal and Russo, 2019). That so few women in Ethiopia have knowledge of both availability and even one legal ground for abortion may help explain why levels of unsafe abortion remain high, despite changes towards the liberalization of legal protections (Gebrehiwot *et al.*, 2016).

Previous qualitative research has highlighted the important role of health-care workers in providing information and access to abortion services (Blystad *et al.*, 2019; Tadele *et al.*, 2019). Our finding of no relationship between knowledge and distance to health facilities that offered contraceptive services or contraceptive and abortion services aligns with the argument that it is only direct contact with the health system and health providers specifically, rather than general availability of services, that affects knowledge. While there was some variation in these relationships in terms of knowing only about legal grounds and only about availability, in general, our results point to a positive relationship between having contact with the health system and having greater knowledge about abortion services and legality. Continued focus on clarifying the legal grounds for accessing abortion services and promoting abortion as a safe and effective means to prevent maternal morbidity and mortality among public health providers, including HEWs—with whom contact was significantly associated with greater knowledge in our study—is an important strategy to encourage the dissemination of accurate information. Additionally, more research on the extent of provider knowledge and resistance to abortion counselling and provision is critical. Recent evidence not only confirms the critical role of mid-wives in serving as sources of information and providers of safe abortion services but also highlights that directive counselling and refusal to provide services are common, underscoring the need to ensure that guidelines on service provision are clear (Fekadu *et al.*, 2022). The importance of providers is additionally particularly relevant to consider in the context of the GGR; recent evidence from Ethiopia suggests that both NGOs (Mavodza *et al.*, 2019), which are generally considered private providers, and the public health system (Holcombe and Kidanemariam Gebru, 2022; Sully *et al.*, 2022; Vernaelde, 2022) were impacted by the GGR. Evidence suggests that there is currently a reliance on NGOs and private health sectors to promote information about and provide abortion services (Blystad *et al.*, 2019; Tadele *et al.*, 2019), but this strategy will likely be hampered by the cyclical nature of US elections and re-enactments of this policy. Efforts to create alternative financial mechanisms to alleviate the impact of the GGR are critical if wider-scale efforts to disseminate information on abortion and alleviate reliance on health-care workers will not be feasible. While these efforts have been negatively impacted by COVID-19 (Cotroneo, n.d.), our findings provide further evidence of their importance to ensuring comprehensive information is available to women.

Previous research has also raised the concern that reliance on health providers is unlikely to address residential and regional inequities, as providers may be more or less willing to provide this information based on both personal and community beliefs (Blystad *et al.*, 2019; Tadele *et al.*, 2019; Fekadu *et al.*, 2022). We observed significant regional variation among both urban and rural women with significantly lower levels of knowledge in smaller, agrarian regions and higher knowledge among women in Tigray. We note that data for this study were collected prior to the onset of civil conflict in 2020, and thus these relationships are likely to have changed in Tigray. Still, these findings reflect that knowledge does vary by region, underscoring that the impact of liberalization of the law at the national level is unlikely to be felt equally across regions. Ensuring that training curricula for all levels of health providers that is offered during initial medical and nursing education, in addition to ongoing in-service training, include standardized information on abortion procedures, safety and legality may be one way to address disparate information at the provider level. Currently, in-service training efforts include Value Clarification and Attitude Transformation trainings to health workers at all levels of the health system. However, additional research may help inform efforts by identifying if reluctance to provide information varies at the regional level or by provider type. Health Management Information Systems, which track both safe abortion services and post-abortion services, can potentially also be used identify facilities with lower than expected caseloads for additional targeting of such interventions.

In urban, but not rural areas, women who were users of modern contraception were significantly more likely to know either a source of abortion services only or a source and legal ground for abortion. As we were not able to account for abortion history, this may reflect some level of reverse causality (i.e. women who received facility-based abortions previously may have been more likely to be offered and use contraceptive methods). Evidence suggests that use of contraception in the post-abortion period is high in Ethiopia (Beyene *et al.*, 2021), however studies are generally restricted to populations from specific facilities or regions, which limit their generalizability (Prata *et al.*, 2011; Abate *et al.*, 2020; Beyene *et al.*, 2021). Some evidence suggests that urban women are more likely to receive abortions than that of rural women (Abebe *et al.*, 2022; Moore *et al.*, 2016), but challenges with abortion measurement and limited updated population-based data make this challenging to confirm. That this relationship holds in urban, rather than rural areas, may also reflect that contraceptive service providers are more likely to provide additional counselling related to legality and service availability in urban areas, relative to more conservative rural areas.

In urban areas, increasing wealth was positively and significantly associated with greater knowledge of either only a legal ground or both a source and a legal ground. On the whole, access to contraceptive and abortion services is significantly better in urban areas, as evidenced by the fact that all women in urban areas lived within 5 km of a facility that offered at least contraceptive services; however, differences in knowledge of service availability by wealth may point to continued disparities within urban environments. As urban centres continue to grow, addressing the needs of the most vulnerable and guaranteeing access to safe and affordable contraception and abortion services is critical, including through expansion of the public health system within urban centres (Duminy *et al.*, 2021). Recent research indicates a preference to access abortion services, including medical abortion services, through the private sector (Blystad *et al.*, 2019). There are continued efforts to expand the role of the private sector in Ethiopia (Bare and Warren, 2021), and the inclusion of medication abortion within these efforts could serve as an important mechanism to expand overall access. However, as private services tend to be more expensive and less likely to be used by the poor or women in rural areas (Shah *et al.*, 2011; Ministry of Health - Ethiopia, Global Financing Facility, World Bank Group 2019), considerable efforts must be taken to reduce the potential for increasing disparities by socioeconomic status.

Our study raises a number additional research and programmatic questions. While we focussed specifically on the relationship with the health system, further research to explore how knowledge of abortion legality and access is shared, such as through social networks, is critical. Additionally, while perceived backlash to broader promotion of the current abortion regulations is frequently noted as a concern, more research exploring the extent to which this is likely to occur and among whom (i.e. religious leaders, political leaders and partners) is needed. Both fields of research can inform efficient strategies to promote knowledge of abortion availability and legality while addressing potential resistance.

This study is not without limitations. The first, as mentioned earlier, is the potential for reverse causality given the cross-sectional nature of the data. Because of known under-reporting biases related to receipt of abortion, we did not attempt to measure women’s previous use of abortion services; thus, we cannot evaluate whether knowledge of service availability and legality is higher among women who have already received an abortion. Additionally, due to a survey programming error, we did not include a question related to availability of abortion services to adolescents, which is available largely on demand, and thus we may not be capturing women’s full range of knowledge. Given the low level of knowledge among women and the general strategy of not promoting information related to abortion legality and availability, however, it is unlikely that inclusion of this ground would significantly change results or interpretation. Finally, due to low levels of knowledge, we were not able to explore whether these relationships differ by each legal ground. Despite these limitations, however, our study has a number of strengths. First, the data are nationally representative of the general population, adding significant value as studies have largely focused on facility-based or highly-specific populations. Additionally, data for both the SDP and household are collected concurrently and within the same geographic areas; linkages of these data allow for improved estimation of the influence of the service delivery environment on reproductive behaviour.

## Conclusion

Our results demonstrate that women are more likely to have at least some knowledge of abortion legality and availability if they visited a health facility or in the case of urban women, if they were visited by a health worker who spoke them about FP. Overall levels of knowledge are low, however, and a reliance on health workers to serve as gatekeepers, when approximately half of women accessed services in the previous 12 months, may slow dissemination of information related to safe abortion, contributing to continued high levels of unsafe abortion. More research is needed on both provider resistance to offering information and effective communication channels to disseminate accurate information. If the current strategy of reliance on health workers continues, it is critical to disseminate accurate information throughout the public health system, particularly when the GGR is not in effect.

## Data Availability

All data used in these analyses are publicly available and can be accessed at https://www.pmadata.org/data/request-access-datasets. DOIs for the specific datasets used in these analyses are included in the reference list.

## Endnotes

1. While we argue that FP services include both abortion and contraceptive services, in Ethiopia, FP is broadly understood to mean contraception only. As such, questions used the term ‘family planning’ when referring to receipt or use of contraceptive services.

## Reflexivity statement

The PMA-Ethiopia team is composed of a diverse group of individuals. In-country field operations are led by two senior Ethiopian public health professionals and supported by male and female field staff. Junior colleagues, both male and female, are specifically encouraged to contribute to the development of research products, including this manuscript. The authorship team includes four females and five males and span multiple levels of seniority. Five of the authors specialize in academic research on reproductive health, two are senior officials within the Ministry of Health and one is a student at Johns Hopkins.
